# Identification of a Musculus Levator Claviculae on Physical Exam: A Case Report and Literature Review

**DOI:** 10.3390/diagnostics15081008

**Published:** 2025-04-16

**Authors:** Eric Smith, Erik Vanstrum, Ashley Kita

**Affiliations:** Department of Head and Neck Surgery, David Geffen School of Medicine, Los Angeles, CA 90095, USA; emsmith@mednet.ucla.edu (E.S.); evanstrum@mednet.ucla.edu (E.V.)

**Keywords:** imaging, anatomical variants, levator claviculae, cleidocervicalis, thoracic outlet syndrome, surgery, neck dissections, physical exam maneuvers, sonography, MRI

## Abstract

**Background and Clinical Significance**: The levator claviculae muscle (also known as cleidocervicalis) is a vestigial muscle located in the posterior triangle of the neck, extending from the upper cervical transverse processus to the clavicle. It has been detected in ~2% of humans, but is rarely documented in the radiologic or anatomic literature. When found on physical exam, it is usually mis-identified as lymphadenopathy, metastasis, cysts, an aneurysm, or other masses. It has been implicated in a few cases of thoracic outlet syndrome. **Case Presentation**: Herein, we describe a 25-year-old man with a weightlifting history, who was found to have a right levator claviculae muscle in the setting of unilateral mixed neurovascular thoracic outlet syndrome. The patient presented with right-sided extremity paresthesias, pain in the neck, shoulder, and arm, and symptom exacerbation with overhead activities. He also described intermittent unilateral pulsatile tinnitus during strenuous exercise. On physical exam, he was found to have a right carotid bruit, unequal systolic blood pressures, and positive Roos and Adson’s testing. The variant muscle was identified with a modified exam maneuver, and was further characterized with sonography and MRI. Symptoms were managed with activity restriction and NSAIDs. We reviewed 17 cases of levator claviculae variant muscles in patients. **Conclusions**: The presence of levator claviculae muscles has been detected in patients with thoracic outlet syndrome, but never in a patient with an audible bruit and pulsatile tinnitus. This physical exam maneuver, used in conjunction with multimodal imaging, successfully aided diagnosis and direct medical management in this case.

## 1. Introduction

Thoracic outlet syndrome (TOS) is a condition involving compression of the neurovasculature structures that supply the upper extremities [1,2]. Compression usually occurs at the interscalene, costoclavicular, and subpectoral minor spaces, and may cause forms of arterial, neurogenic, venous, or mixed TOS [1,2]. The pathology of TOS is highly variable, and may be attributed to a person’s anatomy at the individual level. The most well-known factors include anomalous first and cervical ribs, ligamentous bands, and/or compression and irritation from scalene, pectoral, and/or subclavius muscles [1,2]. Lesser known anatomical muscle variants may also contribute to the pathophysiology of TOS in certain patients, such as the subclavius posticus and levator claviculae muscles [3,4,5,6,7]. Evaluation of TOS involves identifying the structure responsible for compression through physical exam maneuvers and imaging, such as with ultrasound and MRI [2]. Treatments may involve lifestyle modifications, physical therapy, muscle injections, and/or operative decompression [2].

There have been reports of large anatomic muscle variants hypertrophying and leading to compression of the neurovasculature in the supraclavicular fossa between the cervical and thoracic outlets [6,7,8,9]. These muscles likely hypertrophy with other shoulder muscles with usage, but the prevalence of isolated muscle variant hypertrophy is unreported in the literature [10,11]. The most well-known variant is referred to as the subclavius posticus, which is an accessary muscle belly that extends from the upper border of the scapula to the first rib [6,7]. It is believed to branch from the omohyoid, and is estimated to have a prevalence of 4.9% in both cadaveric and MRI studies [4,12]. Another muscle variant, known as the levator claviculae (also known as the cleidocervicalis), is estimated to have a prevalence of 2–3% in the population, but the actual prevalence may be lower [13,14]. The levator claviculae is likely a vestigial respiratory muscle used in primates [15]. Today, its attachment points are debated, but the general consensus is that a true levator claviculae extends superiorly from the cervical vertebrae to the clavicle inferiorly [14,16,17]. In clinical patients, it has not always been possible to trace and confirm its superior attachment point, because this variant muscle can blend with trapezius or levator scapularis fibers [14]. Others have referred to variants of the levator claviculae as a “supraclavius” or “anomalous” muscle in select clinical patients, with its superior fibers blending with the trapezius muscle or extending to the nuchal ligament, respectively [8,9,18]. In this report, we will refer to this muscle and its variants as levator claviculae muscles. Regardless of this superior attachment, hypertrophy of a levator claviculae muscle likely can narrow the thoracic outlet, compressing the neurovasculature [8,9,18]. Its presence of is not always apparent, and there is a lack of a standardized approach to assess for a levator claviculae muscle in patients [8,18]. Given the numerous detections of this muscle in cadaveric studies, the true prevalence of accessory muscles contributing to TOS remains unknown [14].

Herein, we describe a case of a levator claviculae muscle identified in a patient with mixed arterial and neurogenic TOS by using a new physical exam maneuver, in addition to standard imaging techniques. We also review 17 cases of patients with variant levator claviculae muscles from the literature, in order to determine the muscle’s typical presentation and associated symptoms, methods of diagnosis, and types of treatments employed.

## 2. Case Presentation

The patient was a 25-year-old male who participated in upper body lifting and had no other significant prior medical history. He was not taking any prescription medications. He presented with a 5–6 year long history of right-sided extremity paresthesias, described as tingling and heaviness. He also experienced right-sided neck, shoulder, and arm pain, with symptoms further exacerbated with overhead activity. At previous wellness exams, 5 years prior, his providers noted a right carotid bruit and differences in systolic blood pressures (right ~8 points lower). A right carotid ultrasound and cardiac echo resulted in no clinically relevant findings, including no stenosis or narrowing of the carotid artery. Over the next 5 years, the patient experienced two episodes of dual right arm claudication and pre-syncope, precipitated by significant right arm usages. The patient also made note of an audible unilateral right-sided whooshing sound during strenuous exercises. He had temporary improvements in his symptoms by limiting overhead activities and attending physical therapy.

**Figure 1:**
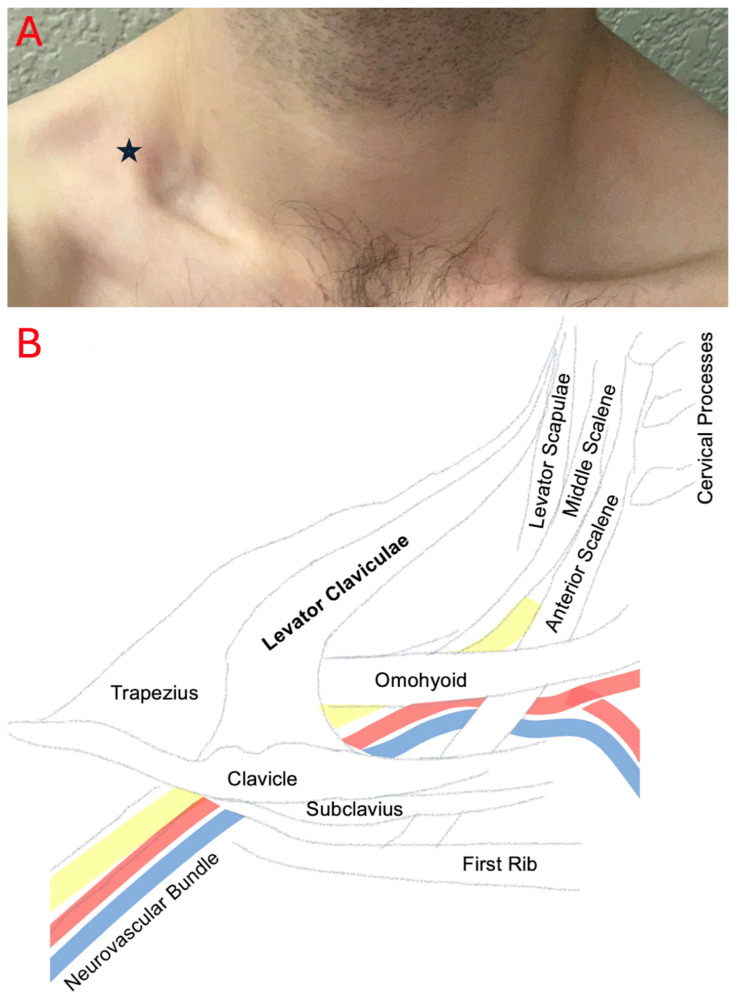
The right levator claviculae muscle (starred) present in the supraclavicular fossa, with attachment to the clavicle inferiorly and extending superiorly towards the trapezius muscles (**A**). A diagram demonstrates the expected anatomy adjacent to the variant muscle (**B**).

The patient sought follow-up care due to incomplete resolution of symptoms with activity restriction, symptom flares precipitated by activity, and a desire for a definitive diagnosis. He was found to have positive Adson’s and Roos tests. These tests, when used in combination, have a sensitivity and specificity of 72% and 82%, respectively [19]. These symptoms and exam maneuvers were consistent with mixed neurovascular TOS. Additionally, an angular deformity of the right mid-clavicle prompted further examination. A large difference in depth of the right supraclavicular fossa was noted compared to the left when the patient elevated his shoulders and upwardly rotated his scapulas. The protrusion felt muscular in nature, and appeared as a strip that could be isolated and traced from the clavicle inferiorly to the trapezius superiorly (Figure 1). Its lower portion appeared lateral to the sternocleidomastoid before reaching the trapezius muscle.

**Figure 2:**
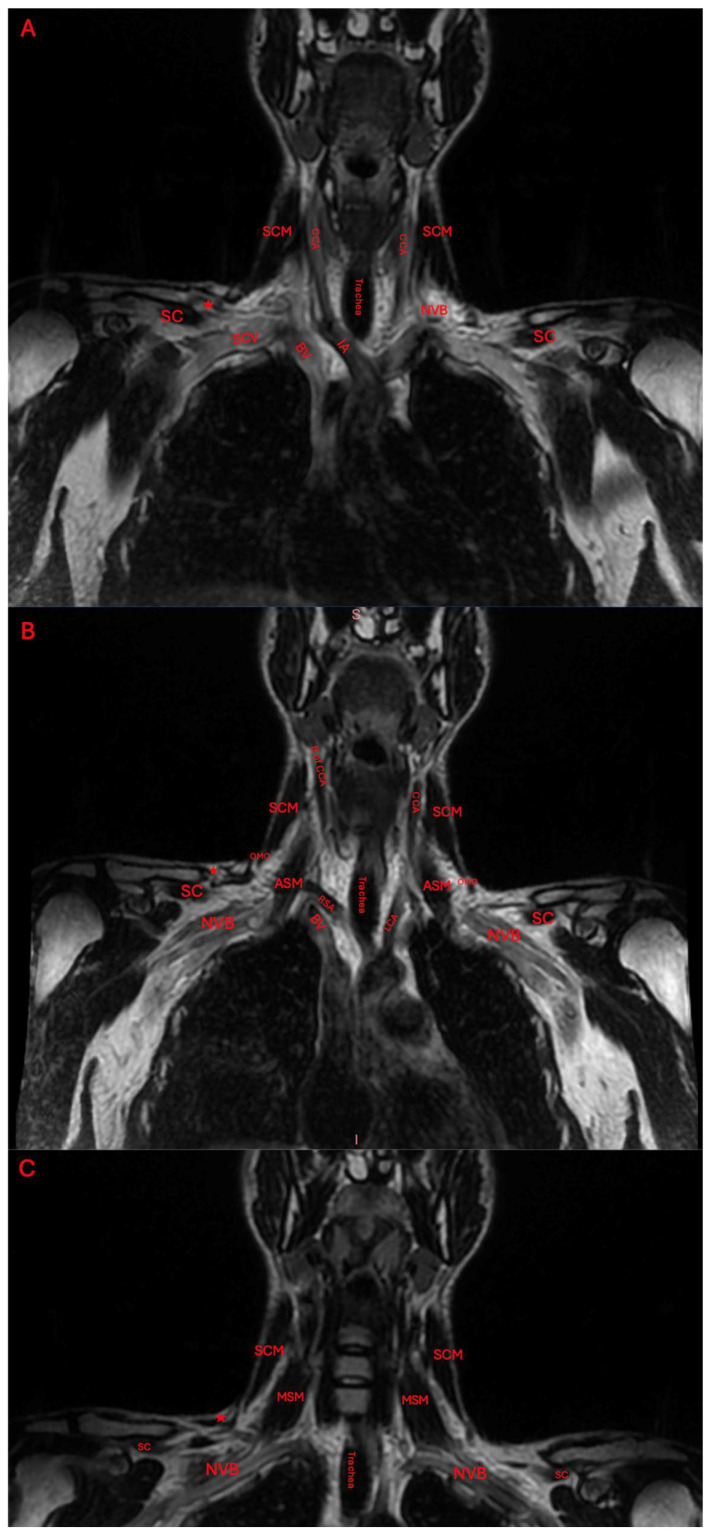
Coronal T2-weighted MRI imaging of the brachial plexus, neck, and shoulder moving from anterior to posterior (**A**–**C**). The red star marks the insertion of the levator claviculae. The other abbreviations are the bifurcation of the carotid artery (B of CCA), left common carotid artery (LCA), innominate artery (IA), sternocleidomastoid (SCM), right subclavian artery (RSA), anterior scalene muscle (ASM), middle scalene muscle (MSM), brachiocephalic vein (BV), omohyoid (OMO), subclavius muscle (SC), and neurovascular bundle (NVB).

**Figure 3:**
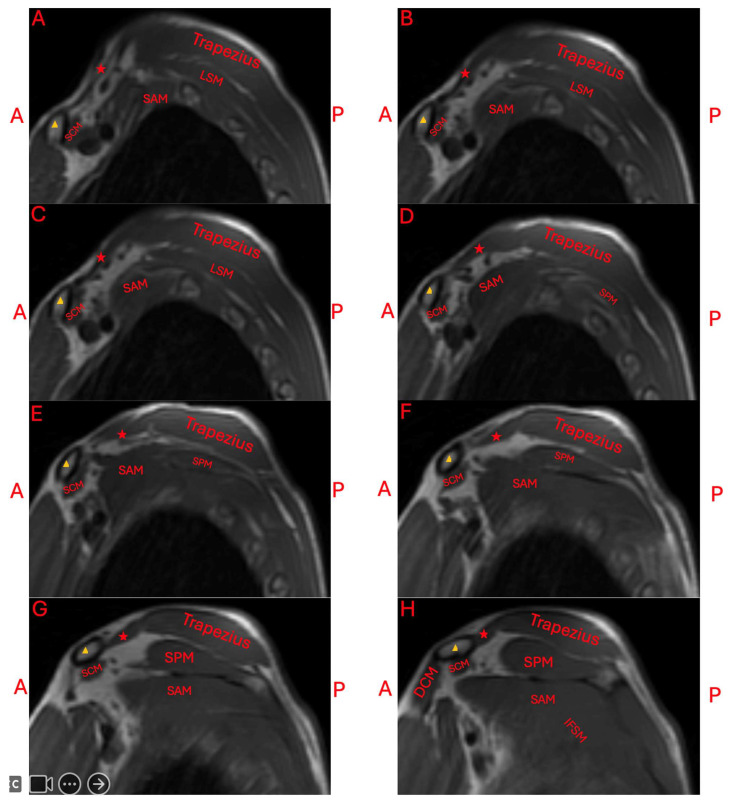
Sagittal T1-weighted MRI imaging of the right shoulder. Progression from the medial to lateral shoulder view can be followed from (**A**–**H**). The images are oriented anteriorly on the left and posteriorly on the right. Red stars mark the levator claviculae muscle, and yellow triangles mark the clavicle. Other muscles include the levator scapulae muscle (LSM), serratus anterior muscle (SAM), subclavius muscle (SCM), supraspinatus muscle (SPM), infraspinatus muscle (IFSM), and deltoid clavicular part (DCM).

The patient was seen by an outside neurologist who performed an ultrasound of the right neck. This neurologist reported the presence of normal skeletal muscle inserting at the clavicle and blending with the trapezius. They additionally reported a cervical band subjacent to the brachial plexus, and a transverse artery traveling between the upper and middle trunks. An MRI was ordered to further characterize the accessory muscle. Its location was found to be superior to the neurovasculature bundle (Figure 2 and Figure 3).

**Figure 4:**
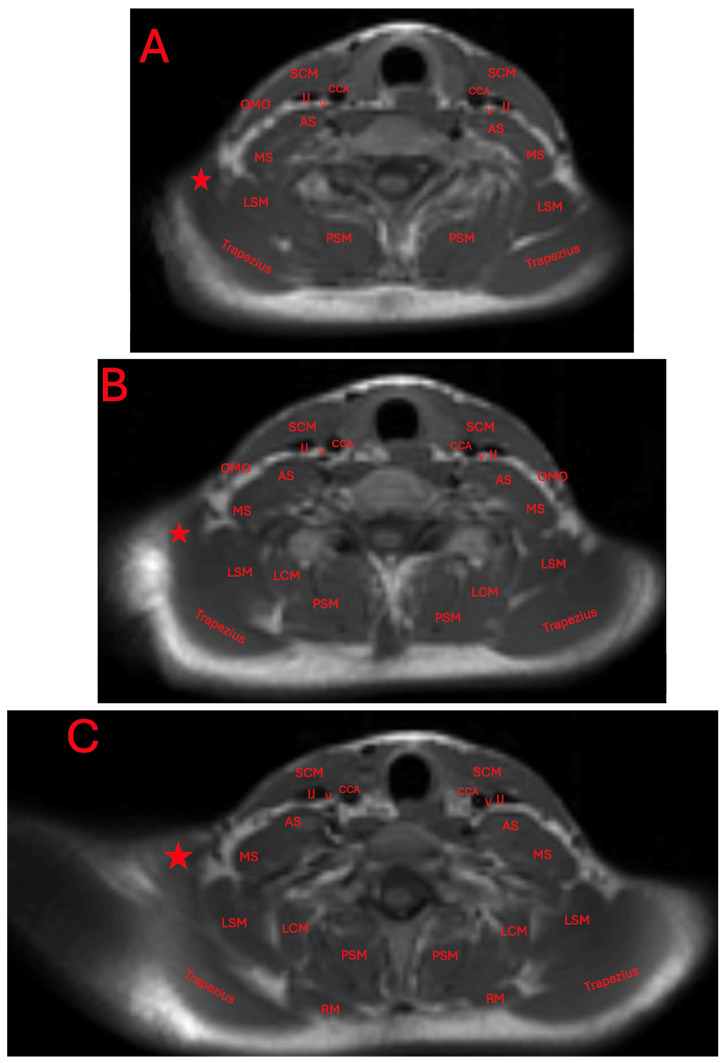

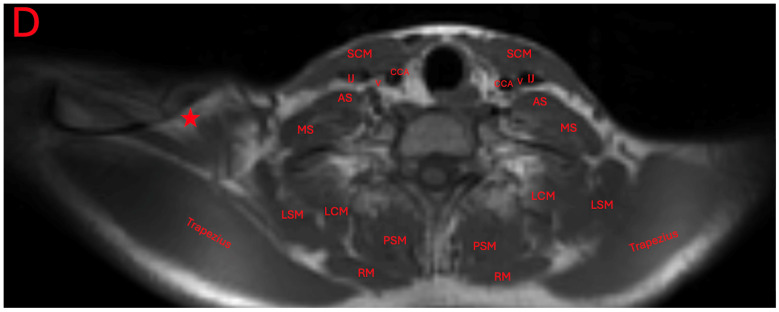
Axial T1-weighted MRI imaging of the patient’s neck and shoulder (**A**–**D**). Red stars indicate the position of the levator claviculae muscle. The other abbreviations are the common carotid artery (CCA), vagus nerve (V), internal jugular vein (IJ), sternocleidomastoid (SCM), anterior scalene muscle (AS), middle scalene muscle (MS), omohyoid (OMO), levator scapulae muscle (LSM), longissimus cervices muscle (LCM), paraspinal muscle (PSM), and rhomboid muscle (RM).

The presence of this accessory muscle can also be observed adjacently to the trapezius and levator scapulae muscles, and attached to an irregular protuberance of the clavicle (Figure 4A–D).

The patient modified his exercise practices to limit further hypertrophy of the shoulder muscles. For painful symptom flares, conservative management was used, which included both ice and NSAIDs. Significant improvement was reported at 1 year follow-up.

## 3. Literature Review

### 3.1. Search Strategy

**Figure 5:**
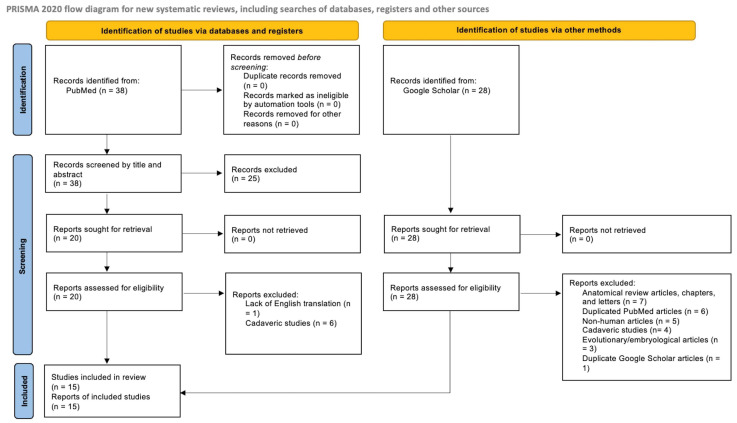
PRISMA 2020 flow diagram protocol using databases (PubMed) and other sources (Google Scholar).

A systematic review to identify known cases of levator claviculae muscles in living patients was conducted, following the PRISMA 2020 guidelines, and the protocol is displayed in Figure 5 [20]. The database search was performed in PubMed in January 2025, using the terms “Levator Claviculae OR Supraclavius OR Cleidocervical Muscle OR Cleidocervicalis OR Musculus Omocervicalis OR Musculus Cleidocervicalis OR Cleidoatlanticus OR Cleidotrachelian”, which yielded 37 results. One reviewer screened the results by examining titles and abstracts, and 12 articles were identified that reported a relevant variant muscle located in the posterior triangle in living patients. The “Recently Cited By” section was also reviewed for these clinically relevant articles, and one additional article was located. An additional search in Google Scholar using the term “cleidocervicalis thoracic outlet” identified 28 results and yielded two additional cases of the variant muscle in clinical cases. Of these 28 results, 6 were duplicate articles identified in PubMed, and thus were excluded. Exclusion criteria for both resources encompassed articles without an English translation, review articles without novel reports of the muscle in clinical patients, and non-clinical reports of the muscle in cadavers, embryos, and animals. We also excluded review articles, books, and letters discussing standard shoulder muscles. There was no time cutoff date for identified reports. This review was not registered on PROSPERO, since the goal was to review reported cases. Identified articles were read in full and are summarized below.

### 3.2. Characteristics of Reported Cases

While the levator claviculae muscle is estimated to occur in approximately 2–3% of the population, there are only 17 reports of the muscle occurring in patients in the literature [3,5,8,9,18,21,22,23,24,25,26,27,28,29,30] (Table 1).

As previously mentioned, the nomenclature for a levator claviculae muscle was varied. The muscle was referred to as a levator claviculae or musculus levator claviculae in 12 of these cases (71%), while 4 cases referred to it as a supraclavius muscle (24%), and 1 as an anomalous muscle. All reports of patients with a supraclavius muscle occurred in patients with intra-operative muscle discovery, without usage of imaging to identify its attachment points [8,18]. Muscle fibers were observed to extend directly from the trapezius to the clavicle in 10 cases (59%), confirmed to attach from the transverse process of the cervical vertebrae to the clavicle in 6 cases (35%), and confirmed to attach from the nuchal ligament to the clavicle in 1 case (6%). Our search was limited by the numerous alternative names for this muscle, making it possible that additional reports of the levator claviculae muscle have been published under other terminology.

Of these 17 cases, 12 muscles were detected in men (71%), and 5 muscles were detected in women (29%). The muscle was identified on the right side in eight cases (47%) and on the left in nine cases (53%). There were no reports of bilateral muscles (0%), even though this is known to exist, based on cadaveric studies [16]. A levator claviculae muscle was identified intra-operatively in 6 of the 17 cases (35%), without prior detection on physical exam or imaging [8,18,21]. It was detected initially on physical exam in seven cases (41%), but only four of these were in patients with symptoms of thoracic outlet syndrome [5,9,22,24,26,28,29]. Of these seven cases, the muscle was confused with swelling or lymphadenopathy in six instances (86%) [5,22,24,26,28,29].

In total, 10 of the cases reported levator claviculae muscles in patients with symptomatic thoracic outlet syndrome (59%), and the other 7 instances of the muscle were identified in asymptomatic patients (41%). Of these symptomatic 10 cases, 4 underwent a larger operation incorporating resection or transection of the muscle (40%), 3 underwent primary muscle resection or transection (30%), 2 managed symptoms with conservative therapy (i.e., activity restriction, ice, or NSAIDs) (20%), and no treatment was reported in 1 patient (10%) [3,5,8,9,18,22,24,25]. Patient outcomes were reported in 5 of these 10 symptomatic cases (50%) [3,5,8,9,24]. Both patients managed with conservative therapy saw improvement in their symptoms [3,24]. Of the patients that underwent primary resection of their levator claviculae muscles, known improvements were reported in two of the three cases [5,9,25]. In the more extensive operation group, one patient experienced 90% improvement at their 1-year follow-up [8]. No negative outcomes were reported for all 10 patients [3,5,8,9,18,22,24,25].

## 4. Discussion

### 4.1. Comparisons to the Literature

Identification of the levator claviculae muscle is not trivial, and has been reported few times in symptomatic patients in the literature [3,5,8,9,18,22,24]. Patient symptoms have varied drastically, from reports of standard neurogenic TOS to mixed arterial and neurogenic TOS, like in our patient [8]. This is the first report of a levator claviculae muscle occurring in a patient with an audible carotid bruit on physical exam and intermittent pulsatile tinnitus. More importantly, this case details the identification of the levator claviculae muscle on physical exam prior to imaging or intra-operative discovery. The significance of this report and other cases is yet to be seen, given that the true prevalence of the levator claviculae muscle in symptomatic patients remains elusive.

### 4.2. Physical Examination to Aid Imaging

**Figure 6:**
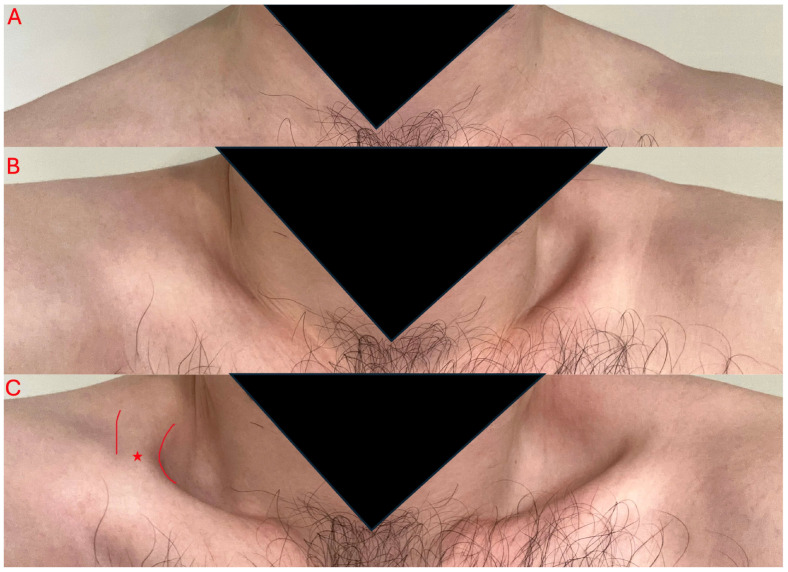
Summit and Valley Maneuver steps, with the shoulders at rest (**A**), shoulders elevated (**B**), and shoulders elevated with simultaneous upward rotation of the scapulas (**C**). A red star and markings outline the observable levator claviculae muscle (**C**).

Reports of the levator claviculae muscle in the literature may have been identified at various states of hypertrophy, or the muscle may have been completely undetectable on gross physical exam. Additionally, the muscle is often not commented on in radiology reports. Therefore, it would be beneficial to be able to identify the muscle on physical exam in symptomatic patients with TOS. Our case is the first to use a standardized physical exam maneuver to identify levator claviculae muscles, which we propose as the “Summit and Valley Maneuver” (Figure 6). (1) Patients would start in a seated or standing position with their arms kept down by their sides (Figure 6A). (2) Next, they would shrug their shoulders upwards (Figure 6B), before (3) upwardly rotating their scapulas forward (Figure 6C). In the vast majority of people, there would normally be an oval depression in the space of the supraclavicular fossa with this movement. Patients with an enlarged levator claviculae muscle may show a steep drop-off adjacent to a strip of muscle that attaches inferiorly to the clavicle and superiorly to the trapezius. The patient may also have an angular deflection located on their clavicle, like in our case and in the report by Ruiz Santiago et al. [28]. This maneuver also provides the opportunity to measure the muscle and/or perform sonography to confirm the presence of skeletal muscle tissue and rule out other pathologies. Sonography is already an accepted practice for diagnosis of TOS, and has been demonstrated to have sensitivity for subtle anatomic variants, such as brachial plexus piercing variations [2,31]. We acknowledge that this maneuver may be limited by extreme body habitus.

### 4.3. Conservative Treatment

Treatment methods for a symptomatic levator claviculae muscle vary in the literature, ranging from conservative to surgical interventions. This is similar to current treatment recommendations for uncomplicated TOS, with escalating levels of care [2]. Activity restriction in our case and in the above cases treated conservatively resulted in an improvement in symptoms. It can be hypothesized that promoting levator claviculae muscle atrophy could decrease compression of thoracic outlet neurovasculature. While physical therapy is beneficial to many TOS patients, patients with variant muscles may have inherent shoulder misalignment, resulting in persistent symptoms, despite recommended therapy visits and ergonomic changes [2,31]. Our case report also documents improvements in subjective symptoms with anti-inflammatory medications. Use of oral NSAIDs improved symptoms in the case presented by Kuiper et al., but risks unwanted gastric and cardiac side effects [24]. NSAIDs are already an accepted therapeutic approach for thoracic outlet syndrome, limiting inflammatory responses secondary to brachial plexus irritation by aberrant adjacent structure [10,32]. There are no current reports of targeted steroid or botulinum toxin injections, but these could be explored in other symptomatic patients [21].

### 4.4. Implications for Surgical Management

Surgical management for thoracic outlet syndrome may include excision of anomalous structures (i.e., cervical ribs, ligamentous bands, etc.), excision of the anterior and middle scalene muscles, resection of the first rib, and/or neurolysis of the brachial plexus [2,33]. Surgeons may approach these structures by a transaxillary, infraclavicular, posterior, and/or supraclavicular approach, with or without endoscopic or video assistance [2,33]. It is highly possible that these approaches (except supraclavicular) fail to identify supraclavicular muscle variants. These variants may also be regarded as typical strap muscles and ignored. Our case suggest usage of conservative management, and the review of the literature has demonstrated that targeted resections/transections of levator claviculae muscles may be a valid option for the management of symptomatic patients with identifiable muscle variants [3,5,9,24,25]. Early identification of a variant muscle can aid surgical planning and direct surgeons to consider smaller muscle resections prior to more extensive rib resections and neurolysis. More extensive TOS operations remain a viable option if patients fail to improve.

## 5. Conclusions

The presence of levator claviculae muscles has been detected in patients with thoracic outlet syndrome, but this is the first report of the muscle being identified in a patient with an audible bruit and pulsatile tinnitus. In addition, this case report details a standardized physical exam maneuver, named the “Summit and Valley Maneuver”, that can be used in conjunction with multimodal imaging for muscle characterization and may guide further management. Future studies would be needed to identify the percentage of TOS patients that have an identifiable levator claviculae muscle. As seen from this case report and the reviewed cases, patients with identifiable levator claviculae muscles have benefited from conservative management and targeted surgical procedures on this muscle.

## Figures and Tables

**Table 1:** The 17 cases of levator claviculae muscles and similar variants previously reported in the literature, along with a summary of our reported case.

Reference	Muscle Referred as	Side	Muscle Attachment Points	Year Reported	Age	Gender	Presenting Symptoms	Diagnostics	Treatment	Outcome
Aydoğ et al. (2007) [3]	Levator claviculae	Right	On physical exam: Extended from medial neck to middle clavicle. On imaging: Lateral to SCM and attached to middle clavicle.	2007	26	Male	Right neck, shoulder, and supraclavicular pain. (+) Roos, (−) paresthesias or fatigue of the arm.	Physical exam, sonography, MRI	Activity restriction, ice	Resumed activity
Ferreli et al. (2019) [21]	Levator claviculae	Left	Intra-op: Attached to C2-C3. Anterior to trapezius and levator scapulae muscles, lateral to scalene muscles, medial to SCM.	2019	61	Male	Asymptomatic, incidental finding.	Intra-op	None	Unknown
Ginsberg et al. (1999) [22]	Levator claviculae	Left	Imaging: Superiorly blended with left paraspinal muscles at C3 level. Posterior to SCM with inferior extension and insertion at mid-aspect of clavicle.	1999	37	Female	Pain radiating to upper neck, with tender “lump” palpated in lower-left neck.	Physical exam, CT	None reported	Unknown
Costa González et al. (2020) [23]	Levator claviculae	Left	Intra-op: Originated in transverse apophysis of axis and inserted on medial third of clavicle.	2020	N/A	N/A	Asymptomatic, incidental.	Intra-op, identified on CT review	None	Unknown
Greeneway et al. (2022) [8]	Supraclavius	Left	Intra-op: Extension from trapezius muscle to medial superior undersurface of clavicle. Exact origin not pursued.	2022	42	Female	Left arm weakness and numbness. Arm heaviness with elevation. (+) Tinnels sign over left supraclavicular area. Refractory to PT.	Intra-op	Supraclavius resection, supraclavicular left scalenectomy	Improvement of 90% at 1-year follow-up
Greeneway et al. (2022) [8]	Supraclavius	Right	Intra-op: Extension from the trapezius muscle to medial superior aspect of clavicle.	2022	45	Female	Fifteen-year history of right arm weakness/numbness, exacerbated by elevated arm activities. TOS diagnosed with vascular US showing subclavian arterial impingement and nonocclusive DVT of subclavian vein. Refractory pain post-rib resection, pectoralis minor release (infraclavicular approach).	Intra-op	Supraclavius transection, anterior scalenectomy	Unknown
Hug et al. (2022) [5]	Levator claviculae variant	Right	Intra-op: Originating from trapezius and attaching to middle third of clavicle.	2000	48	Male	Sharp pain radiating from neck to radial forearm, thumb, index finger. Lower arm and hand paresthesias (worse at night). Right neck “swelling”. Thumb and radial forearm had diminished sensation without muscle wasting. Irritation with Adson, Roos, Halsted. Worsened with physiotherapy.	Physical exam, MRI	Variant muscle resected	Pain-free status post-surgery and return to normal activities
Kuiper et al. (2014) [24]	Musculus levator claviculae	Right	Physical exam: Extension from superior trapezius to middle third of clavicle. Imaging: MRI demonstrated attachment to transverse process of fourth cervical vertebrae.	2014	27	Female	“Swelling” above clavicle. Painful to palpation at insertion point of muscle (middle third of clavicle). Symmetrical strength.	Physical exam, MRI	Activity restriction, NSAIDs	Reduction in symptoms after 2 months
Kimura et al. (2023) [9]	Anomalous muscle	Left	Imaging: Nuchal ligament to middle third of upper clavicle.	2023	40	Male	Dull posterior neck pain and numbness. Pain and tenderness with shoulder drooping. (+) Bakody’s test, (−) for muscle weakness, (−) Adson’s, Wright, Allen, Roos test.	MRI, repeat physical exam, nerve conduction study, CT scan, MRI, neurography	Variant muscle myotomy, activity restriction for one month	Complete resolution of symptoms
O’Sullivan et al. (1998) [25]	Levator claviculae variant	Left	Intra-op: Directly from trapezius muscle with posteromedial insertion along clavicle, lateral to SCM.	1998	36	Male	Brachial plexus injury (pathology possibly related to variant muscle).	Intra-op	Variant muscle transection	Unknown
Rosenheimer et al. (2000) [26]	Levator claviculae (cleidocervical) muscle	Right	Physical exam: Attached to superior border of median clavicle. Imaging: Attached to transverse process of C6 to inferior clavicle. Was lateral to SCM.	2000	Unk	Female	Asymptomatic, neck mass identified on physical exam (concerns of malignancy).	Physical exam, CT, MRI	None	Unknown
Rüdisüli et al. (1995) [27]	Musculus Levator Claviculae	Left	Imaging: Extension from C3 transversus process to lateral end of clavicle.	1995	56	Male	Asymptomatic, identified on CT during lymphoma staging.	CT, follow-up physical exam	None	Unknown
Salehi et al. (2015) [18]	Supraclavius	Right	Intra-op: Medial attachment to deep superior aspect of the clavicle, with extension to trapezius muscle, and was lateral to SCM.	2014	19	Male	Presented with right arm swelling and cyanosis.	Intra-op	Venography and subclavian vein thrombolysis, supraclavius excision, scalenectomy, first rib resection, subclavian vein reconstruction	Unknown
Salehi et al. (2015) [18]	Supraclavius	Right	Intra-op: Extended from trapezius muscle to superior undersurface of medial clavicle, lateral to SCM.	2014	60	Female	Right lower neck pain, numbness and tingling radiating to arm and hand for >1 year. No improvements with physical therapy.	Intra-op	Supraclavius resection, scalenectomy, brachial plexus neurolysis, first rib removal, pectoralis minor tenetomy	Unknown
Ruiz Santiago et al. (2001) [28]	Levator claviculae muscle	Right	Imaging: Supernumerary bundle of cervical muscles (SCM, trapezius, anterior scalene) with clavicular insertion.	2001	18	Male	Asymptomatic, concerns for malignancy. Hard painless mass in supraclavicular region and angular deflection on clavicle palpation.	Physical exam, plain radiography, CT scan, MRI	None	Unknown
Schlarb et al. (2016) [29]	Levator Claviculae	Left	Imaging: Attached posteriorly to SCM and extending to superior clavicle in lower neck.	2016	14	Male	Asymptomatic, neck mass on physical exam.	Physical exam, CT with contrast	None	Unknown
Shaw et al. (2004) [30]	Levator claviculae	Left	Imaging: Posterior to SCM and extended to clavicle.	2014	24	Male	Asymptomatic, identified on CT for ameloblastoma surgical planning.	CT	None	Unknown
Our Case	Levator Claviculae	Right	Directly from trapezius with posteromedial insertion along clavicle.	2025	25	Male	Right neck, shoulder, and supraclavicular pain, fatigue of right arm, (+) Roos and Adson’s. Audible right carotid bruit, intermittent unilateral pulsatile tinnitus.	Physical exam, sonography, MRI	Activity restriction, and ice and anti-inflammatory medications as needed	Control of symptoms

## Data Availability

The data presented in this study are available on request from the corresponding author, due to privacy restrictions.

## Abbreviations

The following abbreviations are used in this manuscript:

C1-6	Cervical vertebrae 1-6
NSAIDs	Non-steroidal Anti-Inflammatory Drugs
SCM	Sternocleidomastoid
TOS	Thoracic outlet syndrome
